# Recruitment of Occipital Cortex during Sensory Substitution Training Linked to Subjective Experience of Seeing in People with Blindness

**DOI:** 10.1371/journal.pone.0023264

**Published:** 2011-08-10

**Authors:** Tomás Ortiz, Joaquín Poch, Juan M. Santos, Carmen Requena, Ana M. Martínez, Laura Ortiz-Terán, Agustín Turrero, Juan Barcia, Ramón Nogales, Agustín Calvo, José M. Martínez, José L. Córdoba, Alvaro Pascual-Leone

**Affiliations:** 1 Department of Psychiatry, Facultad de Medicina, Universidad Complutense, Madrid, Spain; 2 Department of Ear, Nose and Throat (ENT), Hospital Clínico Universitario San Carlos, Madrid, Spain; 3 CIBERSAM (Centro de Investigación Biomédica en Red de Salud Mental, Spanish Research Network in Mental Health), Madrid, Spain; 4 Department of Psychology, Universidad de León, León, Spain; 5 Department of Radiology, Massachusetts General Hospital, Harvard University, Boston, Massachusetts, United States of America; 6 Department of Statistics, Facultad de Medicina, Universidad Complutense, Madrid, Spain; 7 Department of Neurosurgery, Hospital Clínico Universitario San Carlos, Madrid, Spain; 8 Vision-Tactile Project, Facultad de Medicina, Universidad Complutense, Madrid, Spain; 9 Berenson-Allen Center for Noninvasive Brain Stimulation, Department of Neurology, Beth Israel Deaconess Medical Center, Harvard Medical School, Boston, Massachusetts, United States of America; 10 Instituto Guttman de Neurorehabilitació, Universitat Autònoma de Barcelona, Barcelona, Spain; National Institute of Mental Health, United States of America

## Abstract

Over three months of intensive training with a tactile stimulation device, 18 blind and 10 blindfolded seeing subjects improved in their ability to identify geometric figures by touch. Seven blind subjects spontaneously reported ‘visual qualia’, the subjective sensation of seeing flashes of light congruent with tactile stimuli. In the latter subjects tactile stimulation evoked activation of occipital cortex on electroencephalography (EEG). None of the blind subjects who failed to experience visual qualia, despite identical tactile stimulation training, showed EEG recruitment of occipital cortex. None of the blindfolded seeing humans reported visual-like sensations during tactile stimulation. These findings support the notion that the conscious experience of seeing is linked to the activation of occipital brain regions in people with blindness. Moreover, the findings indicate that provision of visual information can be achieved through non-visual sensory modalities which may help to minimize the disability of blind individuals, affording them some degree of object recognition and navigation aid.

## Introduction

Cross-modality sensory stimulation may offer a good opportunity to improve recognition, localization and navigation in blind people. A coherent and unified perceptual experience is created by the brain with multisensorial input [1]–[3], although the neural substrate of this multimodality integration is not yet fully understood yet. Some areas of the brain, mainly the lateral occipital cortex, are specialized for visual object recognition and can be activated by tactile stimuli [4]–[6]. This activation of the visual cortex might lead to visual-like perception, regardless of the sensory input modality [3].

In the blind the high demand required by object recognition appears to also recruit ventral and dorsal occipital areas [7]. Blindness modifies neocortical processing of non-visual tasks, including frontoparietal and visual regions during tactile stimulation [8]. Some authors have reported sources of connections in bilateral primary somatosensory cortex as well as in inferior temporal areas during tactile stimulation in blind subjects, and have also found that the age of onset of blindness could affect the set of areas involved [8]. It is also known that people with blindness proficient in the use of a visuo-tactile sensory substitution device that presents visual images as patterns of electric stimuli to the subject's tongue [9]–[10] show occipital cortex activation in an orientation-discrimination task [10].

Evoked potentials are useful to study brain activity triggered by external stimuli using surface recordings. Around 50 msec after stimulation, and even earlier, primary somatosensory cortical activation is normally found. In the case of tactile stimuli, this happens in parietal areas contralateral to the stimulated hand. Circa 100 msec shape and object automatic recognition takes place as well in somatosensory areas. After 300 msec some activity related to objects with poor-defined identity happens, as well as some matching between perception and stored representations in cognitive processing in prefrontal areas [11]–[17]. As in the case of imaging studies, ERP-based results suggest that tactile inputs could produce occipital cortex activation in the blind [18].

However, there are no published studies aimed at understanding the relationship between activation of lateral occipital cortex and the ability to recognize objects presented to the hand along time. Thus, we tested if repetitive passive tactile stimulation leads to activation of visual areas and recognition of spatial patterns in people with blindness.

We used passive repetitive tactile stimulation over a period of 3 months, one hour a day for five days a week, with vertical, horizontal and oblique lines generated randomly by a tactile stimulator. Our aims were (a) to study if repetitive tactile stimulation can create cross-modality and improve recognition and localization of patterns in blind people, and (b) to evaluate the impact of this training on brain activity. We performed EEG recording during the initial stimulation session and in the last session.

## Materials and Methods

### Subjects

After a call for volunteers was made, we studied 18 blind subjects (13 men, 5 women) that were recruited consecutively from the National Organization of Spanish Blind to participate in the study, plus10 controls (blindfolded seeing normal individuals, 4 men and 6 women) of similar ages to the blind group, who had no visual deficit at all and had no previous history of synesthesias. All participants were informed of the nature and purpose of the experiment and all gave their written informed consent. Whenever minors were consented, both the minor and his/her legal representative were informed, minors' assented and their legal representatives consented in written form acknowledging the minors assent as well. The study was approved by the Hospital Clínico San Carlos Clinical Research Bioethical Committee (Universidad Complutense, Madrid, Spain) committee/institutional review board and consent adhered to the Declaration of Helsinki. Participants had no history of neurological, psychiatric, cognitive or sensorimotor deficits, other than blindness. They all had normal EEGs at baseline.

Nine subjects had early onset blindness (loss of vision before 5 years of age) while the rest, but one, were late blind, having lost their sight after age 15. Four subjects had absolute congenital blindness (from birth), ten had no residual vision at all at the time of our study, while eight had minimal residual vision. Causes of blindness were diverse: congenital nystagmus, glaucoma, retinopathy, congenital cataracts, lenticular fibroplasia, macular degeneration, optic atrophy, Peter's anomaly with microphtalmia, retinal detachment, retina necrosis, retinitis pigmentosa and uveitis (Table 1).

**Table 1:** Sample detailed description.

*Subject*	*Age*	*Sex*	*Handedness*	*Cause of blindness*	*Onset of blindness (years)*	*Residual Vision*	*Subjective perceptions of vision*
1	16	F	R	Congenital nystagmus	1	Yes; light perception <5%	Yes: “I can see a white line … Honestly, I can see it but I do not feel it”
2	14	M	L	Retinitis pigmentosa	4	Yes; light perception <7%	Yes: “I can see white lines … I can see lights oriented either vertically, horizontally or obliquely … I see it but I do no longer feel it in my hand”
3	15	F	R	Bilateral uveitis	4	Yes; light perception <7%	Yes: “There are lines in grey tones, steel-like”
4	32	M	R	Retinal detachment	12	No	Yes: “I see phosphenes always oriented in the direction of the line … I see phosphenes, but can hardly feel it on the hand”
5	51	M	L	Microphtalmia and Peter's syndrome	3	Yes; light perception <3%	Yes: “I can see a luminous line on the dark screen … A major change happened when I suddenly completely forgot the tactile sensations and started seeing in my mind the lights transmitted by the stimuli”
6	69	M	R	Bilateral uveitis	35	No	Yes: “I can see light with greater and greater clarity and intensity”
7	72	F	R	Optic pathway bilateral atrophy	9	No	Yes: “It is like if I see aligned lights”
8	22	M	R	Retrolental fibroplasia	from birth	No	No
9	40	M	L	Retinal necrosis	30	No	No
10	43	M	R	Glaucoma and retinopathy	32	No	No
11	50	M	R	Optic pathway bilateral lesion	22	No	No
12	56	F	R	Macular degeneration and retinosis	29	No	No
13	57	M	R	Macular degeneration and spot degeneration	45	No	No
14	59	M	R	Congenital cataracts	From birth	Yes; light perception <7%	No
15	42	M	R	Congenital glaucoma	From birth	No	No
16	31	M	R	Retinitis pigmentosa	16	Yes; light perception <3%	No
17	64	M	R	Congenital cataracts	From birth	Yes; left eye: light perception <2% (right eye: no)	No
18	54	F	R	Optic pathway bilateral atrophy	1,5	Yes; light perception left eye <2%, light perception right eye <7%	No

M = male, F = female. R = right, L = left. L/P = light perception.

All of the blind subjects were initially considered as a single category, but they were later classified into two groups according to the subjective report, or its absence, of visual-like perception. Group 1 (G1, seven blind participants who reported visual sensations, mean age = 32.83, SD = 15.78); Group 2 (G2, eleven blind who did not and even denied visual sensations when explicitly asked, mean age = 46.99, SD = 13.03); and Group 3 (G3, ten blindfolded subjects with no visual deficit, mean age = 39.12, SD = 11.17).

All but three subjects were right-handed and were stimulated on the palm of their right hand. The left-handed individuals underwent the procedure on their left hands (video S1, supplementary material).

### Stimuli

All subjects underwent intensive training in the use of a tactile sensory device. The stimulation program was completed over a period of three months and involved daily sessions five days a week (Monday to Friday). The control group (G3) underwent exactly the same protocol but they were blindfolded just before each training session until the session ended.

Training consisted of repetitive tactile stimulation with lines oriented vertically, horizontally or obliquely in a random fashion, using a tactile piezoelectric device. Each line was presented for 300 msec at 40 Hz, and followed by a blank pause of 700 msec; hence each direction repeated during one second. The frequency of 40 Hz was chosen based upon the findings that thalamo-cortical connections fire at 40 Hz [19]–[20].

Each stimulation session lasted 60 minutes and included the presentation of approximately 3600 stimuli per session. The generation of repetitive passive tactile stimuli was achieved using a single tactile piezoelectric device with 1536 stimulation points (i.e., a tactile matrix with 32×48 pixels). Each nylon point had a 1.3 mm diameter and they were spaced every 2.4 mm, equally in both dimensions. The device generated a 5 cms double line made up 30 dots each (60 dots in total). The dots of each line were stimulated simultaneously, not directionally. Each point was individually controlled both in height (.0 or 0.7 mm) and frequency (40 Hz) by a custom developed software program.

### Technique

EEG was recorded with a 32 channel Neuronic Medicid Equipment using a standard 10–20 electrocap. We used 32 channels (Fz, pFz, Cz, pCZ, Pz, Oz, Fp1, Fp2, F3, F4, F7, F8, PF3, PF4, pC3, C4, PC4, T1, T2, T3, T4, T3A, T4A, T5, T6, P3, P4, O1 and O2) from the standard 10–20 electrocap. Impedance of all electrodes was kept below 5 kΩ. The electrooculogram (EOG) was recorded with two pairs of leads in order to register horizontal and vertical eye movement. Data were recorded using a mastoid electrode as reference. Sampling rate was 1000 Hz. Amplifier frequency bands were set between 0.05–30.0 Hz.

### Source localization

**Figure 1:**
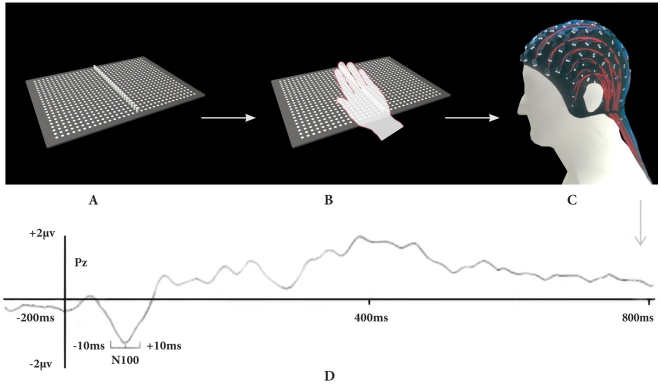
Experimental protocol and analysis of N100 component in Pz electrode. (A) A tactile piezoelectric device with 1536 stimulation points of stimulation is used (B) on the dominant hand, being applied on the palm. (C) EEG is then recorded with a 32 channel-cap. (D) The electrophysiological response of the evoked potential N100 component was measured in each task. Only frequent stimuli responses were analyzed. The time frame to analyze the N100 component was 80–140 ms and it was determined by searching for the maximal amplitude in the respective time window at the Pz electrode. The LORETA analysis was made opening a time window of −10 to +10 ms starting from the high amplitude pick measured in Pz electrode.

Low-resolution electromagnetic tomography (LORETA) was applied to each individual event-related potential (ERP) recording to identify underlying brain electric sources of the scalp potentials. LORETA is a reverse solution method that computes the three-dimensional distribution of neural generators in the brain as a current density value (A/m2), for a total of 2394 voxels, with the constraint that neighbouring voxels show maximal similarity. This analysis was realized for a time window of 20 msc (between −10 and +10 msc starting from the high amplitude peak measured from the Pz electrode) (Figure 1). Brain volume anatomical restrictions were applied for cerebral electrical tomography (CET) calculations and an average brain template was used. CET data were analyzed in the time domain and tomography was calculated for each instance separately.

Source analysis under model (anatomic construction) was obtained using the traditional LORETA method [21]. Different models were defined by constraining the source to one anatomic compartment, chosen using the probabilistic brain atlas (PBA) [22]–[23] and Brodmann atlas.

### Behavioral and EEG measurements

Subjects were instructed to simply experience the stimulation and try to identify the line orientation. Subjects were not allowed to move their hand during the stimulation (video S1, supplementary material).

Both at baseline and the end of the tactile training program, all subjects were tested using a two-alternative forced-choice response task and underwent an ERP study.

For the behavioural assessment, subjects were asked to discriminate pairs of tactile stimuli: horizontal (80% of the stimuli, frequent) or oblique lines (20%, infrequent). The latter task required blind subjects to first detect each stimulus; they were then asked to exclusively respond to the oblique lines by pressing a button. Subjects' responses, errors and response times were collected for further analysis.

For the ERP study, EEG recording was performed in a soundproof room with dim lighting. Subjects were comfortably seated and were instructed to stay awake, keep their eyes open and avoid abrupt movements. Only non-target trials (horizontal lines) were considered for ERP N100 latency (N100) analysis in order to avoid contamination with motor neural activation during response production. Epochs were 1000 msec in duration with a 200 msec pre-stimulus interval and a post-stimulus length of 800 msec. Baseline was defined as the average voltage over the period from 200 msec prior to stimulus onset.

### Data analysis

In order to establish if there were significant differences in reaction time (RT) and N100, before and after training (pre-test and post-test) in each group, paired t-tests were used. Linear regression analyses and Pearson correlation coefficients were carried out to examine the relationships between pre-test and post-test values of the variables RT and N100 in each group. Evaluation of differences between groups due to the training was done using a repeated-measures mixed-effects model. The between-subjects factor was the group (G1, G2 and G3) and the within-subjects factor was the time while the repeated measures were the RT and N100. Statistical analyses were conducted using SPSS and Statgraphics Plus software.

Source analysis for each participant was carried out (Figures S1, S2 and S3, supplementary material). G1, G2 and G3 means (LORETA) were calculated for baseline (pre-test) and final evaluation (post-test), respectively. In each group, voxel-by-voxel Statistical Mapping (SM) was computed to find the activated sources mean differences between pre-test and post-test. Dependent Hotteling 2 test for multiple comparison of pre-test and post-test within each group and independent Hotteling 2 test for inter-groups comparison (G1 vs G2, G1 vs G3 and G2 vs G3) within each state were used. In both instances these parameters were considered: degrees of freedom and threshold values for α<0.05, <0.01 and <0.001).

## Results

### Behavioural results

The behavioural results demonstrate that over three months of tactile stimulation training, blind participants (G1 and G2), as well as the blindfolded seeing controls (G3), demonstrated an increase in tactile discriminative ability. Before training, G1 and G2 subjects made an average 15% of omission errors (failures to recognize the tactile stimuli) and reported incorrect line orientations in an additional 7% of stimuli (identification errors). At the end of the experiment, there were on average less than 1% omission errors and less than 1% identification errors in G1 and G2. Average omission errors before training in G3 (blindfolded control group) were 17% while at the end of the experiment errors were reduced to 2%.

During the last three weeks of the experiment seven individuals (G1) reported that they were seeing lights or brief flashes of light (phosphenes) while undergoing the tactile stimulation. These phosphenes appeared to become elongated into lines and eventually appeared vertical, horizontal or oblique, consistent with the tactile stimuli presented. In almost all cases, the perceived visual sensations matched the presented tactile stimuli (<1% mistakes within G1) and appeared to become more and more vivid with time. At this stage an open narrative account was taken from this group who experienced visual qualia. Moreover, the vividness of the visual percepts eventually was spontaneously reported as much greater than the tactile sensations, which appeared to lose saliency in four of these seven subjects (Table 1). None of G2 or G3 subjects reported phosphenes or any other visual-like sensations.

As it can be inferred from Table 1, no obvious pattern, either epidemiological or clinical, can distinguish those blind subjects who experienced visual qualia (G1) from those who did not (G2), except their age of onset of blindness (Table 1, 9.8 vs 16.15, respectively). It is worth noting the four individuals suffering from absolute blindness from birth belong to G2, as well as most of late onset of blindness subjects. On the contrary G1 includes several subjects with no residual vision and several who had become blind in early childhood.

### ERP and RT results

N100 and RT means and standard deviations, pre-test and post-test, for each group are shown in Table 2.

**Table 2:** Pre-test and post-test comparisons of N100 and RT in each group.

	Pre-test mean (SD)	Post-test mean (SD)	t-statistic	p value
N100				
G1	120.3 (6.8)	91.9 (7.3)	3.012	0.024*
G2	105.1 (5.4)	98.3 (5.9)	0.686	0.508
G3	98.8 (5.7)	101.7 (6.1)	−0.413	0.689
RT				
G1	645.5 (29.5)	616.5 (27.7)	1.492	0.186
G2	606.3 (23.5)	559.9 (22.1)	1.716	0.117
G3	643.5 (24.7)	620.5 (23.2)	2.167	0.058

Paired t-tests results and p-values are shown. (*) statistically significant.

Paired t-tests shows statistically significant differences only for N100 in G1 (p = 0.024), that is to say, N100 latency in G1 decreased significantly because of training.

**Figure 2:**
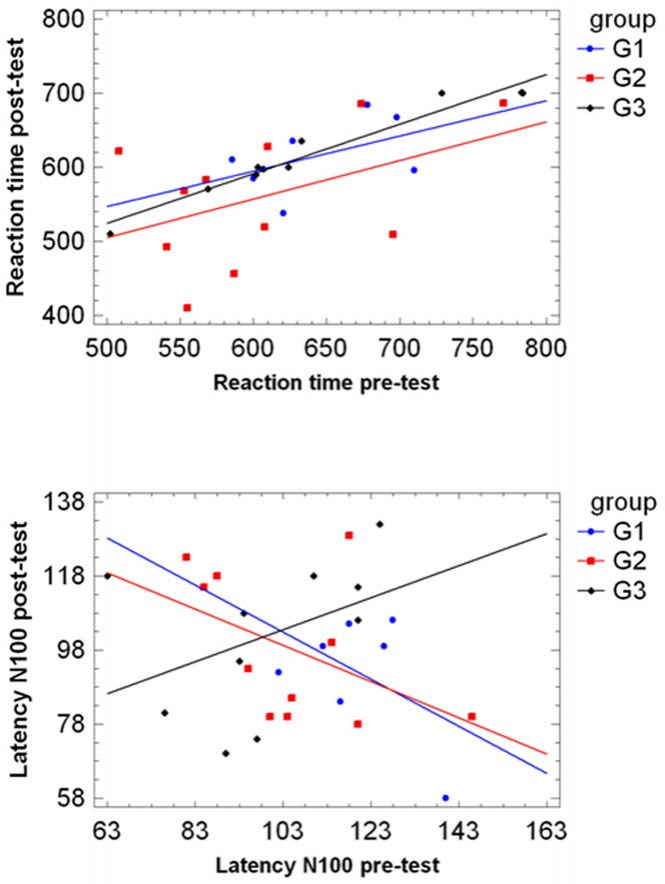
Regression lines of RT (top) and N100 (down) post-test values vs. their respective pre-test values.

The relationship between pre-test and post-test values for N100 and RT was investigated using linear regression analyses in the three groups. Figure 2 shows the results of these findings. In both G1 and G2, a negative trend is observed for N100. There is an inverse correlation between pre-test and post-test N100 values indicating a tendency for decreased N100 post-test when N100 pre-test increases, and viceversa. G3, on the contrary, shows an opposite pattern since the pre-test and post-test N100 values are positively correlated. However, this effect is not statistically significant, according to a t-test of the slope of the regression line, in all groups (all p>0.14), with correlation coefficients r = −0.47 in G1 and G2 and r = 0.42 in G3.

The results for the variable RT are more homogeneous in all groups as the correlation is positive in all of them, but not statistically significant in G1 and G2 (both p>0.16) with correlation coefficients r = 0.47 and r = 0.45 respectively. However this effect is statistically significant in G3 where a strong linear relationship is observed between the pre-test and post-test RT values (p<0.0001) with a correlation coefficient r = 0.98.

**Figure 3:**
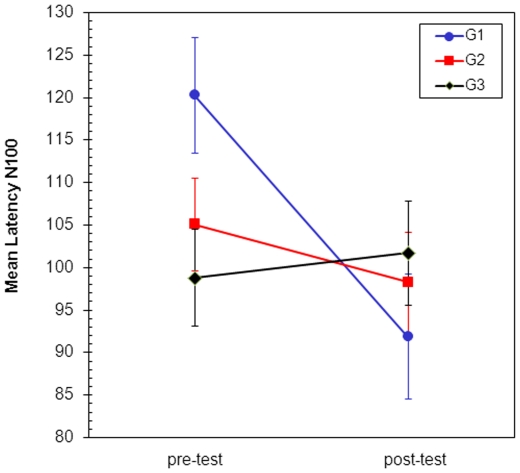
Standarized means plot of N100 scores. Error bars indicate SE.

To evaluate the effects of training, a repeated-measures mixed-effects model was used. Figure 3 shows the means plot for N100. The three groups exhibit a divergent behaviour along time, which suggests a possible interaction N100*group effect.

Statistical analysis shows differences close to statistical significance amongst N100 means along time (F = 4.12, p = 0.053). The interaction N100*group is also close to being statistically significant (F = 2.70, p = 0.087). Finally, the group effect is not significant (F = 0.45, p = 0.641).

Training effect upon N100 was previously studied in every group separately (Table 2). Group effect analysis is rather futile as G1 and G2 did not have similar N100 start-offs. The interaction N100*group is very important for our study and it is close to statistical significance. It seems the possibility of repeating this analysis with a larger sample size could be very useful.

**Figure 4:**
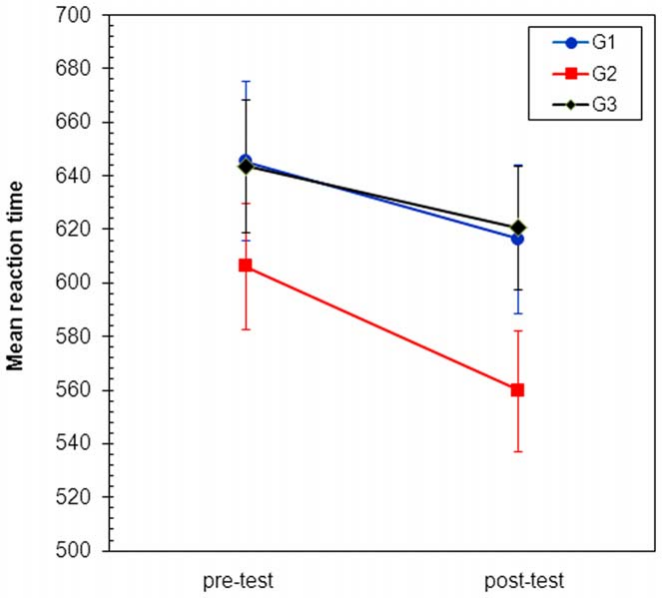
Standarized means plot of RT scores. Error bars indicate SE.

Figure 4 shows the means plot for RT. The three groups exhibit a similar behaviour along time so there was no apparent RT*group interaction. There are significant differences between RT means along time (F = 6.82, p = 0.015) and the interaction RT*group is not statistically significant (F = 0.36, p = 0.702). Again, the group effect is not significant (F = 1.68, p = 0.207). The first result shows that, as a whole, the training effect is significant. The previous analysis using paired t-test for each group, separately, showed no significant differences (all p>0.05).

### Source localization

**Figure 5:**
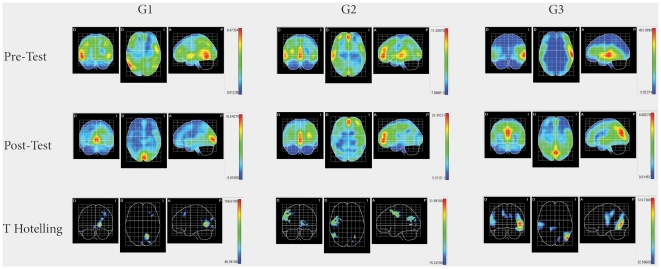
Two upper rows show cortical intensity projection (LORETA) mean maps obtained in pre-test and post-test in each group. Maximal intensity projection areas are displayed in red. **Lower row show Statistical Mapping (SM) dependent Hotelling T^2^ significant differences maps between pre-test and post-test in each group.**

As illustrated in Figure 5, between pre-test and post-test, people with blindness who experienced visual-like percepts have a shift in maximal intensity projection cortical areas after repetitive tactile stimulation, from temporal-parietal (auditive, tactile) to occipital (visual) ones, between pre-test and post-test. In fact, the greatest activation in G1 pre-test is found exclusively in temporal areas (right inferior temporal, right mid-temporal lobes and left superior temporal). G1 post-test maximal activation is identified in bilateral calcarine and lingual areas. On the other hand, G2 pre-test maximal activation is seen in frontal middle orbital, medial superior frontal and anterior cingular areas, all of them bilaterally, as well as right superior and middle temporal areas. G2 post-test is found to be in middle orbital and medial superior frontal areas, both bilaterally, as well as in bilateral anterior cingulate cortex. G2 does not show any occipital pole activation whatsoever. G3 pre-test maximal activation is localized in the left middle and superior temporal areas and does not show any occipital pole activation. G3 post-test maximal activation is found in the cunneus, precunneus and calcarine areas, all of them bilaterally.

Using SM significant differences between pre-test and post-test for each group are found in G1 in BA 17, 18, 19, 9 and 46, in G2 in BA 4, 9 and 39, and in G3 in BA 21, 22, 7, 17, 18 and 48 (Figure 5). Clusters of significant voxels, as well as a brief description of the anatomic localization, are summarized in Table 3.

**Table 3:** Summary of maximal intensity projection areas significant differences, with each specific localization, for each Group in pre-test and post-test (dependent T^2^ ).

						Mean	
AAL	BA	x	y	z	T^2^ Hotelling	Pretest	Postest
G1							
Left Calcarine	18	108	76	152	166,6682**	1,9642	5,3346
Left Calcarine	17	109	76	151	150,82 87 *	1,7493	4,6792
Left Lingual	19	110	75	152	134,9632 *	1,7117	4,5672
Left Lingual	18	107	72	150	130,4462 *	1,7647	4,7350
Left Occipital Superior	19	115	99	176	72,6548 *	1,7124	5,0543
Right Middle Frontal	9	48	125	80	61,7910 *	0,7742	0,7643
Left Frontal Middle	9	128	120	70	59,0519 *	1,4497	0,4701
Right Lingual	17	85	65	176	50,7121 *	2,4206	11,8244
G2							
Right Frontal Middle	9	44	128	80	29,0635**	0,5201	1,6203
Right Angular	39	44	97	144	21,8607 *	1,2949	2,5158
Left Superior Occipital	17	100	90	192	20,4368 *	0,9870	0,6192
Left Calcarine	16	96	69	196	18,9137 *	0,8518	0,8928
Right Post-central	3	46	108	107	17,1995*	0,8363	1,1732
G3							
Left Middle temporal	37	114	72	160	124,7300***	7,7973	1,7731
Left Middle occipital	19	136	79	161	112,1210***	90,6255	2,3392
Left Inferior Occipital	37	140	61	154	100,2741***	211,7078	2,4962
Left Inferior Temporal	37	144	63	155	105,7695***	201,8498	2,7039
Left Fusiform	37	131	57	145	67,1131**	103,5997	1,6238
Right Opercular Rolandic	48	31	87	107	59,3316**	118,1168	2,2864
Right Postcentral	43	24	95	105	57.8739**	72,0106	1,1750
Right Superior Temporal	48	35	79	107	56,8068*	143,7001	2,5432
Right Middle Cingulum	23	73	115	127	50,2679**	3,3344	1,2536
Left Angular	39	131	102	150	46,4443**	5,4738	0,8514
Right Middle Cingulum	23	70	116	128	45,7150*	1,4218	0,5232
Right Supramarginal	40	26	110	110	43,6336**	26,9931	1,2630
Left Superior occipital	18	108	104	159	36,3355**	6,1381	4,9371
Left Cuneus	18	108	106	164	33,9557**	7,7053	5,3461
Right Middle Temporal	20	35	59	102	22,7929*	127,5687	1,7227
Right Sup Parietal	5	72	143	142	22,8391*	17,9683	2,0495
Right Inferior Parietal	40	35	116	140	21,8265*	9,9151	1,7221
Right Post-central	3	46	108	107	17,1995*	0,8363	1,1732
Right Inferior Temporal	20	36	32	92	17,5265*	15,0795	0,3774

G1 T^2^ (3–5) = 147.284 for α = .01,  = 46.383 for α = .05. G2 T^2^ (3–10) = 28.466 for α = .01,  = 15.248 for α = .05. G3 T^2^ (3–9) = 72.40764 for α = .001,  = 32.59783 for α = .01,  = 16.76635 for α = .05. AAL = Anatomical label corresponding to Probabilistic Brain Atlas. BA = Brodmann areas. x, y, z = co-ordinates from PBA in three spatial axes. L = Left; R = Right. * p<.05; ** p<.01 ** and p<.001***.

**Figure 6:**
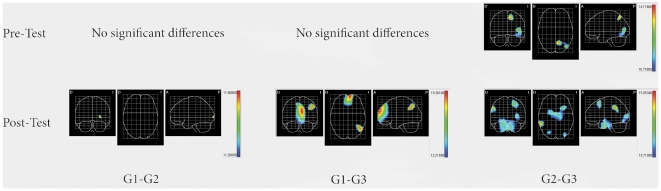
Cortical projections of inter-group comparisons in each state: significant differences maps between groups. LORETA mean maps obtained with independent Hotelling T^2^.

Figure 6 illustrates significant differences inter-groups. There are significant differences in pre-test between G2 and G3, but not between G1 and G2 or G1 and G3. There are also significant differences in post-test between all the groups (G1 and G2, G1 and G3, G2 and G3). During the pre-test, those significant differences happen in BA 5, 7, 19 and 37. With regards to the post-test, significant differences between G1 and G2 are in BA 17. Between post-test G1 and G3, differences happen in BA 39, 32, 11 and 10. Finally, significant post-test differences between G2 and G3 are in BA 40, 39, 36, 35, 19 and 18. Significant differences between groups either in pre-test or post-test are summarized in Table 4.

**Table 4:** Summary of maximal intensity projection areas significant differences, with each specific localization, inter-groups in each state (independent T^2^ ).

AAL	BA	x	y	z	T^2^ Hotelling	Mean	
*Pre-test*						*G2*	*G3*
Left Precuneus	7	104	120	148	14,1740*	1,4213	5,6151
Left occipital middle	19	136	79	171	12,4028*	1,4846	50,0659
Left Precuneus	5	105	130	156	12,2543*	1,9200	7,1426
Left Occipital Inferior	19	131	60	160	11,1081*	1,7229	22,5433
Left Fusiform	37	124	63	153	11,0257*	1,0918	39,8153
*Post-test*						*G1*	*G2*
Left Occipital Middle	17	120	76	188	11,5080*	2,8626	1,779
*Post-test*						*G1*	*G3*
Left Angular	39	131	108	139	19,3610*	1.0899	1,0510
Left Frontal Superior	10	104	95	22	18,1697*	0,6452	0,4443
Left Inferior parietal	39	132	112	144	17,0643*	2,9607	2,7202
Right Middle Superior	10	87	101	33	16,8351*	1,8370	2,0047
Left Middle Superior Frontal	10	104	81	36	15,4223*	0,7925	0,6370
Left Anterior Cingulum	32	88	87	48	14,0570*	3,5205	3,2940
Left Frontal Sup Orbital	11	104	63	29	13,8279*	2,1197	1,3116
Right Orbital Middle Frontal	11	87	63	32	13,1320*	3,8556	3,2801
Right Anterior Cingulum	32	87	77	48	13,0165*	3,3313	2,9261
*Post-test*						*G2*	*G3*
Right Suparmarginal	40	24	112	132	15.8530*	0,4546	0,8095
Left Middle Frontal	8	116	131	72	12,5649*	6,3213	0,9366
Left Fusiform	36	114	35	97	12,2738*	0,3167	1,6700
Right Parahippocampo	35	72	45	90	12,1686*	0,7467	1,0636
Left Middle Occipital	18	120	76	174	12,1421*	1,3117	0,7835
Right Angular	39	29	101	141	12,0550*	3,8171	2,4069
Rigt Fusiform	18	64	60	167	11,9019*	1,8244	1,6919
Right Middle Tempo Pole	36	64	37	86	11,8748*	0,6551	0,8717
Rigt Lingual	18	68	62	167	11,2649*	1,9821	1,9651
Left Hippocampo	35	112	53	99	11,1020*	0,3501	1,5751
Rigt Fusiform	36	66	29	91	11,0176*	0,5722	0,7780
Righ Inferior Occipital	19	56	59	168	11,0393*	2,2506	1,5684

Pre-test G2 vs G3 T^2^ (3–19) = 10.7186 for α = .05 . Post-test G1 vs G2 T^2^ (3–15) = 11.4105 for α = .05. Post-test G1 vs G3 T^2^ (3–14) = 12.21604 for α = .05. Post-test G2 vs G3 T^2^ (3–19) = 10.7186 for α = .05. AAL = Anatomical label corresponding to Probabilistic Brain Atlas. BA = Brodmann areas. x, y, z = co-ordinates from PBA in three spatial axes. L = Left; R = Right. * p<.05; ** p<.01 ** and p<.001***.

## Discussion

After tactile passive repetitive training during an extended (3 months) intensive period people with blindness who spontaneously report visual sensations (G1) exhibited a much shorter RT, considerably decrease in N100 latency and, in parallel with their visual sensations, an activation of the occipital lobe. However no activation of the occipital cortex was found in those blind subjects who did not report any visual qualia (G2), the subjective experience of seeing. We postulate that activation of the occipital cortex, by tactile stimuli results in visual qualia in some blind subjects. Functional activation of the visual cortex by non-visual tactile stimulation has been reported in people with blindness [18], [24]–[33], or with normal vision [17]. Our finding is in tune with other researchers who have stated that massive plasticity in visual cortex for the blind for tactile object recognition does happen [7], [34], [35]. Thus novel evidence linking activity in early visual cortex with visual qualia is provided [33]. Specifically, we show that extended tactile training for several months leads to cross-modal stimulation of occipital cortex and visual-like perception.

It seems as if subjects in G1 ‘learned’ a synesthetic perception with repeated stimulation. It is rather interesting that in G1 subjects tactile sensation decreased when the visual qualia became prominent (Table 1), consistent with maximal activation of visual areas and lack of activation of parietal tactile areas. G2 and G3 subjects experienced exclusively tactile sensations throughout the experiment, again consistent with parietal lobe activation. Thus persistent tactile sensation (at subjective level) seems to preclude the experience of phosphenes. Our study supports the idea that sensory input in one modality can give rise to a percept in another sensory domain, and that the latter can attenuate the subjective percept of the former through training.

There was a frontal activity in G2, both in pre-test and post-test, which did not happen in the other two groups. An increased attentional process may have played a role and, in fact, the four blinds from birth within G2 had a very marked frontal activity (Figure S2, supplementary material). Previous literature [36] has suggested congenitally blind humans exhibit middle temporal cortex (hMT+) and occipital recruitment. Our study replicates the hMT+ finding, yet all blinds from birth in our study belonged to G2 and failed to show occipital activation or experience visual qualia.

In two groups (G2 and G3) the training program did not elicit changes in the pattern of EEG activation or the subjective experience of the stimulation. One possible explanation for this finding that would account for the disparity between the group memberships (i.e., blind subjects vs. normal seeing controls), takes into account the stability of brain networks including the primary visual cortex. Indeed, whenever there has likely been a well established neuronal network, such as in subjects with late blindness, or complete lack of visual input because of congenital blindness, cross-modal connectivity failed to occur. The issue of congenitally blind still remains controversial, as our findings (blind subjects from birth did not experience visual qualia and their occipital areas were not activated) are not fully concordant with previous research [37].

In normal subjects (G3) at baseline many areas were greatly activated and they were widely distributed, but the pattern of activation (and the number of significantly activated brain regions) was reduced after the training protocol. Though habituation may have played a role in this shift in controls, the initial novelty of line orientation information provided in a non-visual fashion to otherwise normally sighted people may be another explanation for the initial high activation. G3 post-test results may indicate a more efficient way of dealing with tactile stimulation through learning. In sighted subjects, early recruitment of primary visual cortex for tactile processing has been found over a few days of blindfolding and intensive tactile training, or auditory training [7], [38]–[44].

In all groups we found that the pre-test activation of the temporal middle complex was significantly reduced after extended training (post-test). Temporal areas are linked to auditive perception, highly enhanced in people with blindness. Moreover, blind subjects display a higher activation of multiple sensory cortical areas than sighted people [45]. A possible explanation for this phenomenon is that tactile training may refine the way the tactile signal is processed through a more efficient brain organization. Therefore, it may functionally deactivate the temporal areas as these may not serve spatial processing. In our study this happens within a wide distribution of ages, which points towards the idea that brain re-organization in blind people has no expiry date, contrary to what has recently been stated by other researchers [46].

In contrast to previous literature [37], we found that people with congenital blindness may be less capable of cross-modality. On the other hand, our results are consistent with previous studies in reporting visual cortex activation after tactile information in early sight deprivation [10], [24], [25]–[27], [37], [47]. Our results also replicate that occipital activity patterns elicited by tactile inputs are clearly more prominent in subjects with early blindness than in late onset of blindness [25], [48].

Our findings in blind subjects who experienced cross-modal induction of visual percepts (G1) also replicate the “visual” matching of the shape of the stimulus [10]. These G1 subjects had an increased activity in striate areas after the training program was completed. Given the fact these areas were not activated at the beginning, it is reasonable to speculate that some form of neuroplasticity may have taken place. Other authors [49], using a two-session auditory-visual and visuotactile training, have hypothesized that promoting efficient functional development of the ventral pathway in the absence of vision can create vision in the brain. Recruitment of visual areas for non-visual tasks is most likely mediated through increased cortico-cortical connectivity [10], [29], [50]. In G1 all subjects but one had early onset of blindness while G2, with one exception, was made of subjects with either late onset of blindness or congenital one. This might mean that, in most cases, tactile training induced cross-modal neuroplasticity in visual areas and that this neuroplasticity would require some, although not excessive, previous stimulation of the visual pathway.

Other authors [28] have stated that haptic and visual neuronal networks may be shared, at least in those familiar issues, as in the case of lines in different orientations after thousands of stimuli. We have also found that other extrastriate areas participate in the recognition of spatial orientation (Table 3, Fig. 5) [51]. Previous literature [52], [53] suggested that, despite occipital cortex volume reduction, blind subjects have visual cortex activation following somatosensory input guided tasks, consistent with re-organization of the visual cortex afferent and efferent neural pathways [54]. We found this to be the case in blind subjects who experienced cross-modal visual qualia (G1), but not in those fail to do so (G2). On the other hand, normal blindfolded seeing controls (G3) have occipital cortex activation during tactile stimulation. This poses the question whether there is a multimodal brain network for spatial information, independent of the sensory modality input. Some authors have suggested primary visual cortex may be part of this multimodal brain network in congenitally blind subjects [38].

Taking into account evidence from human electrophysiology, neuropsychology and animal studies, some authors have suggested that the brain integrates congruent different sensory modalities and that this integration starts as early as 100 ms after stimulus presentation [55]. The N100 component of tactile evoked potentials is an early peak, thought to arise from activation of primary somatosensory cortex [55], [56]. The shortening of the N100 latency we found in G1 presumably reflects increased co-ordinated firing of thalamo-cortical ensembles of neurons in response to the tactile stimuli [57]. On the other hand, a reduction in N100 latency as a consequence of repeated stimulation has been described with increased familiarization with the physical characteristics of a stimulus [58]. Moreover, the greatest improvement starting with greater N100 latency values happen in G1 (Fig. 2). This also points in the direction previously mentioned that the functional network of G1 subjects may be less efficient at pre-training level, perhaps due to lesser stimulation in earlier life, but it is far more plastic. Perhaps this reduction in N100 latency might help to improve the performing in recognition percept, something that could be particularly useful for blind people in high-speed motion targets. In our view, the simplest explanation is that, at the beginning of tactile stimulation, blind subjects activate a broader range of available neuronal circuits to compensate for their visual loss. As passive repetitive tactile training continues, the brain in the person with blindness re-organizes itself by tuning the input towards areas more efficient in spatial processing, prominently occipital cortex.

A few caveats are in order. First, we used a limited set of EEG channels because this was the only one available for us at the time. Second, it remains to be established whether the nature of tactile stimuli or the intensity and repetitive training fashion are critical for obtaining the observed effect. Third, other neuroimaging techniques, such as resting fMRI, default state or coherence analysis, for example, could throw more conclusive data on the newer neuroplasticity. Another limitation is the restriction to a 3-month training period. We do not know if further changes may occur should the passive tactile training continue for a longer duration. Importantly, the most relevant findings are derived from post-hoc analysis. Therefore, no predictive analysis of regression or otherwise could be run. Finally, we studied a limited sample. This is particularly critical in the case of the four subjects included in our study who suffer from congenital blindness as the previous organization of visual cortical columns may be a limiting factor in this domain. Future studies should address, with a larger sample, which blind individuals are more likely to experience visual qualia and to what extent these qualia may affect spatial-processing.

## Supporting Information

Figure S1 **Maximal cortical intensity projection in each Group 1 (G1) subject.** Cortical intensity projection (LORETA) mean maps obtained in pre-test and post-test in each subject.
(TIF)

Figure S2 **Maximal cortical intensity projection in each Group 2 (G2) subject.** Cortical intensity projection (LORETA) mean maps obtained in pre-test and post-test in each subject.
(TIF)

Figure S3 **Maximal cortical intensity projection in each Group 3 (G3) subject.** Cortical intensity projection (LORETA) mean maps obtained in pre-test and post-test in each subject.
(TIF)

Video S1 **Blind volunteer subject undergoing EEG testing during the passive tactile training during week 12.** At the end of the interview the principal investigator (Prof Ortiz) asks him, in Spanish, about his experience while his hand was stimulated. Sub-titres translate into English the conversation between both.
(M4V)
