# Supplementary figures and images for: Recruitment of Occipital Cortex during Sensory Substitution Training Linked to Subjective Experience of Seeing in People with Blindness
Source: PLoS One. 2011 Aug 10;6(8):e23264. doi: 10.1371/journal.pone.0023264 (PMC3154329; doi:10.1371/journal.pone.0023264)

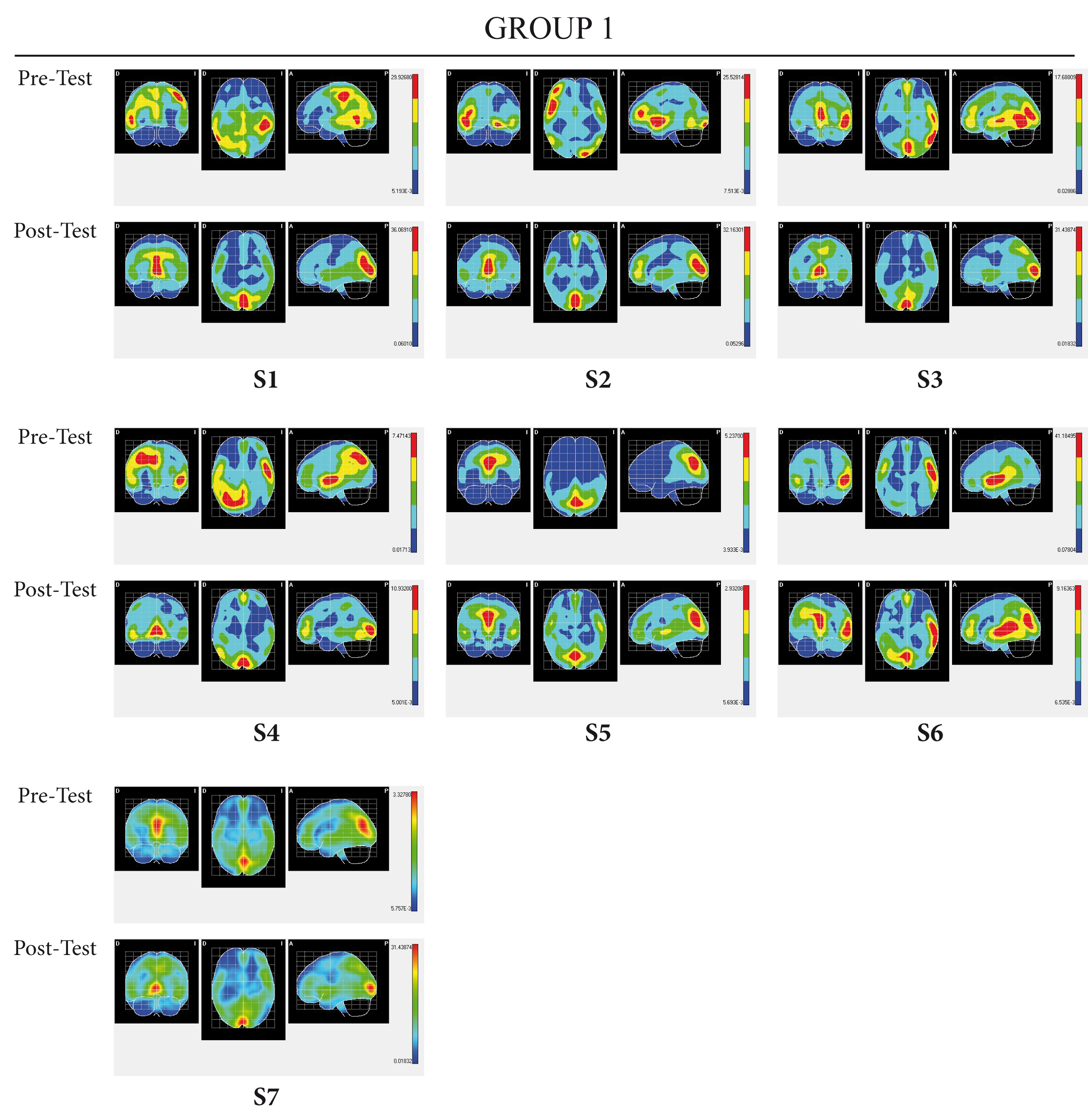

Supplement: Figure S1 — Maximal cortical intensity projection in each Group 1 (G1) subject. Cortical intensity projection (LORETA) mean maps obtained in pre-test and post-test in each subject. (TIF) [file pone.0023264.s001.tif]

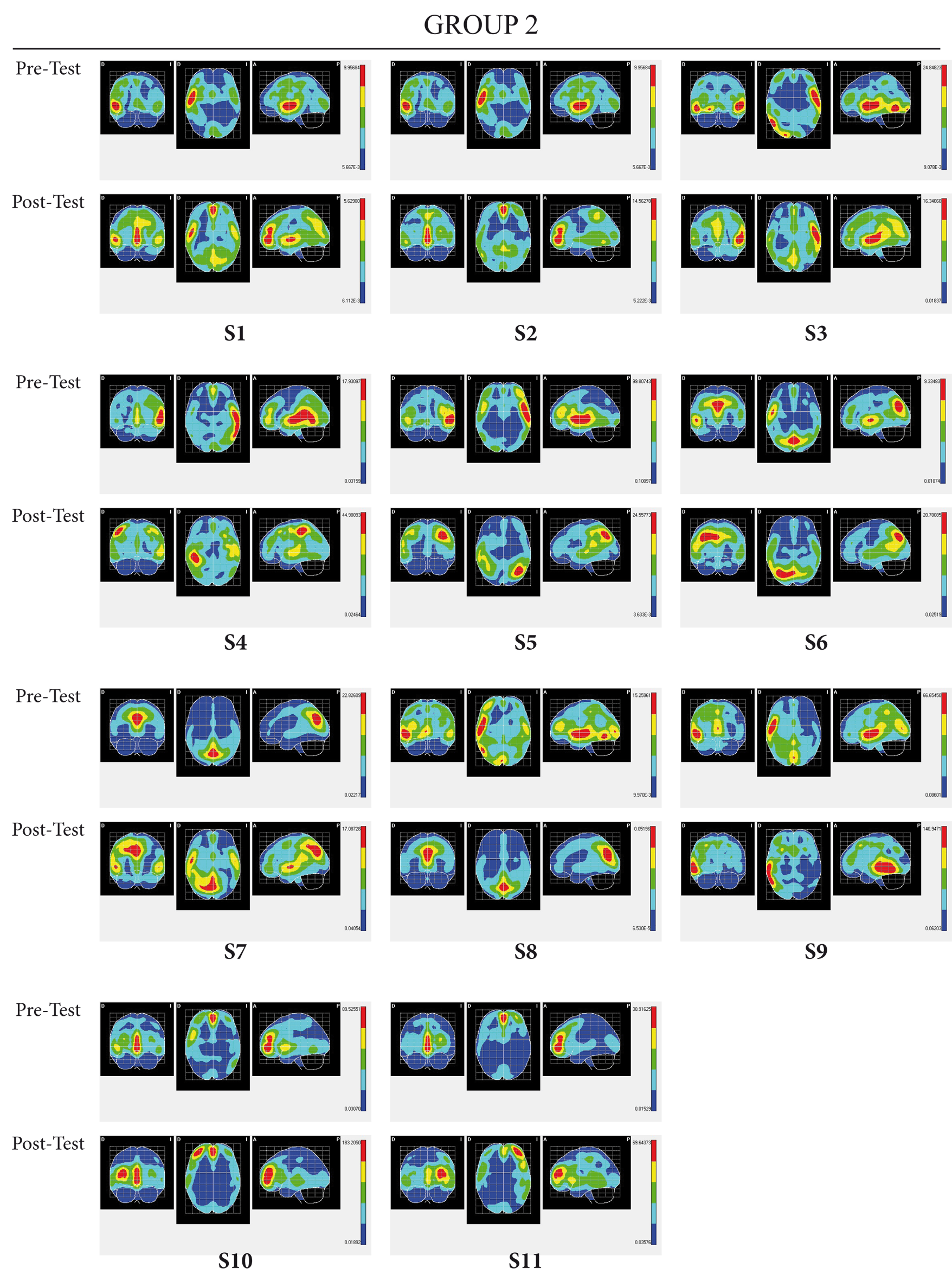

Supplement: Figure S2 — Maximal cortical intensity projection in each Group 2 (G2) subject. Cortical intensity projection (LORETA) mean maps obtained in pre-test and post-test in each subject. (TIF) [file pone.0023264.s002.tif]

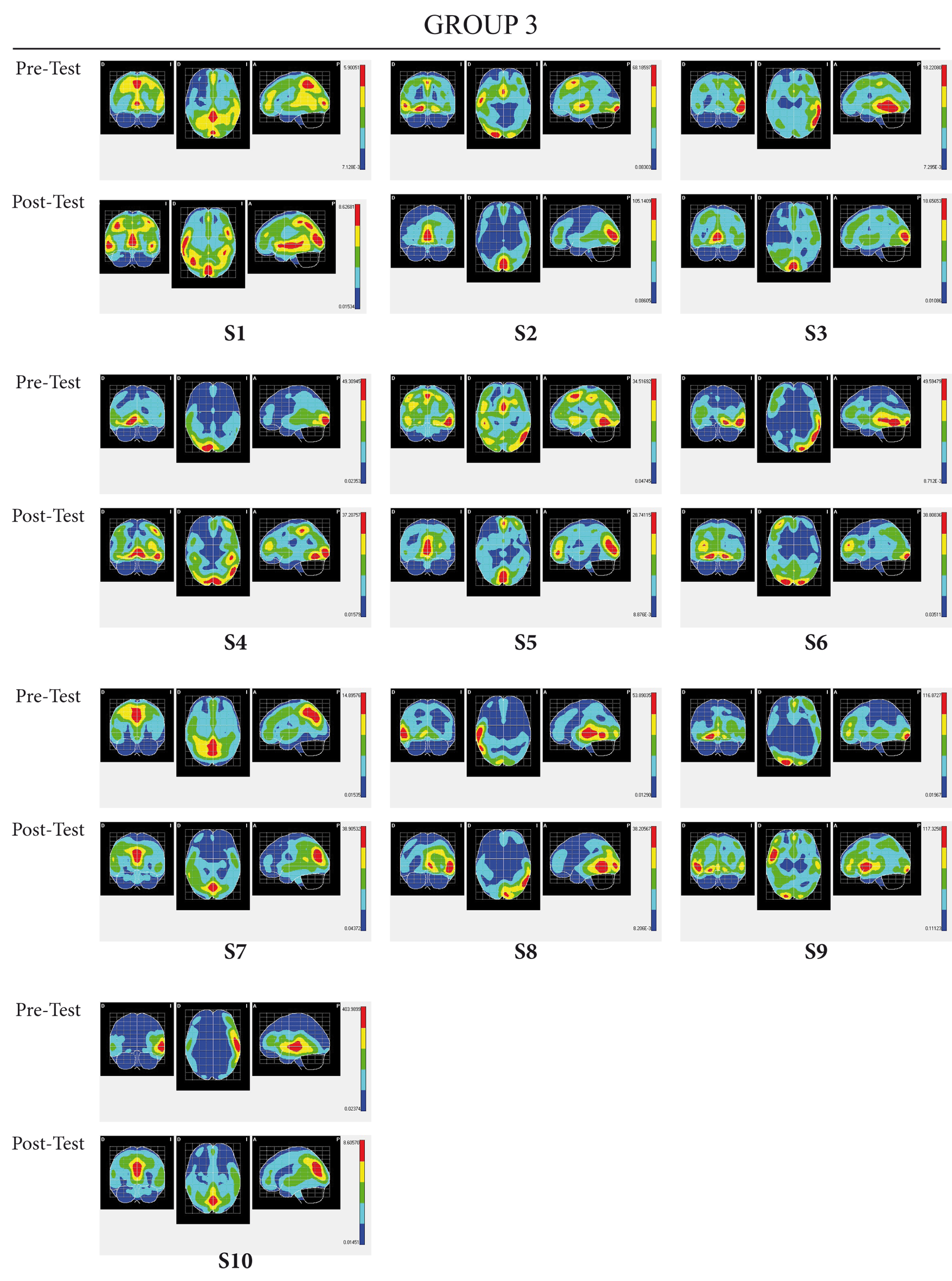

Supplement: Figure S3 — Maximal cortical intensity projection in each Group 3 (G3) subject. Cortical intensity projection (LORETA) mean maps obtained in pre-test and post-test in each subject. (TIF) [file pone.0023264.s003.tif]
